# Cancer-associated fibroblasts and prostate cancer stem cells: crosstalk mechanisms and implications for disease progression

**DOI:** 10.3389/fcell.2024.1412337

**Published:** 2024-07-18

**Authors:** Haoran Chen, Suping Fang, Xudong Zhu, Hao Liu

**Affiliations:** Guang’anmen Hospital, China Academy of Chinese Medical Sciences, Beijing, China

**Keywords:** prostate cancer stem cell, cancer-associated fibroblast, crosstalk, organoid, prostate cancer

## Abstract

The functional heterogeneity and ecological niche of prostate cancer stem cells (PCSCs), which are major drivers of prostate cancer development and treatment resistance, have attracted considerable research attention. Cancer-associated fibroblasts (CAFs), which are crucial components of the tumor microenvironment (TME), substantially affect PCSC stemness. Additionally, CAFs promote PCSC growth and survival by releasing signaling molecules and modifying the surrounding environment. Conversely, PCSCs may affect the characteristics and behavior of CAFs by producing various molecules. This crosstalk mechanism is potentially crucial for prostate cancer progression and the development of treatment resistance. Using organoids to model the TME enables an in-depth study of CAF-PCSC interactions, providing a valuable preclinical tool to accurately evaluate potential target genes and design novel treatment strategies for prostate cancer. The objective of this review is to discuss the current research on the multilevel and multitarget regulatory mechanisms underlying CAF-PCSC interactions and crosstalk, aiming to inform therapeutic approaches that address challenges in prostate cancer treatment.

## Graphical Abstract

**Figure:**
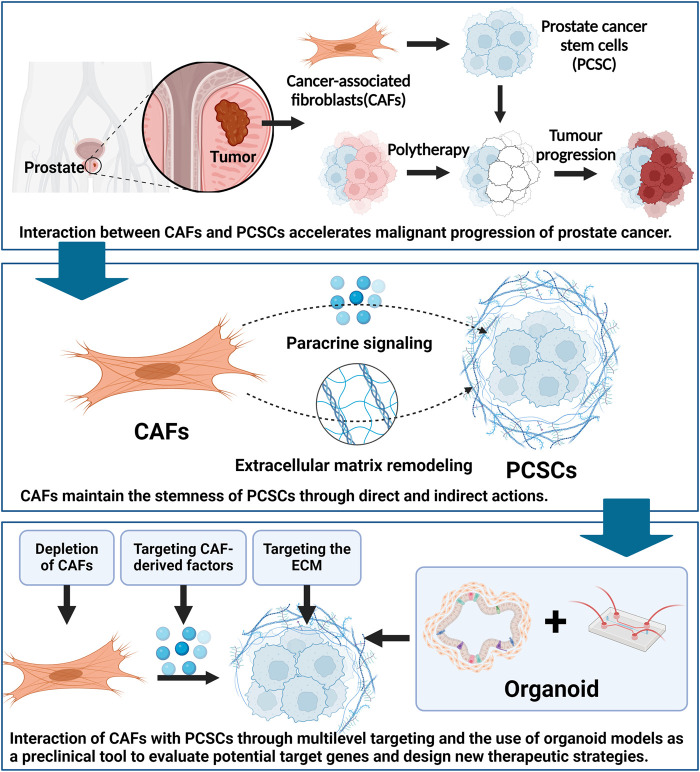
Created using Biorender.com.

## 1 Introduction

In men, prostate cancer (PCa) is the second most common malignancy and the fifth most significant contributor to cancer-related mortality (Bray et al., 2024). Although localized PCa is associated with more favorable outcomes, metastatic PCa remains incurable (Iván et al., 2021). Various therapies, including docetaxel chemotherapy, novel androgen receptor signaling inhibitors (ARSIs), poly (ADP-ribose) polymerase (PARP) inhibitors, and radionuclide therapy, have been approved by the United States Food and Drug Administration (FDA) to treat metastatic PCa (Derya et al., 2024). However, therapy-refractory fatal PCa commonly can develop following the administration of numerous therapeutics (de Wit et al., 2021), leaving the 5-year survival rate of patients with metastatic PCa at approximately 35% (Siegel et al., 2024). Therefore, a detailed investigation of the mechanisms underlying therapeutic resistance in PCa may help to develop effective treatment options.

Recent evidence indicates that extended therapy for patients with PCa may lead to diverse clonal selection (Cheng et al., 2022) and stemness (Verma et al., 2020), ultimately resulting in the development of castration-resistant prostate cancer (CRPC). CRPC is characterized by continued PCa progression and drug resistance following castration therapy via surgical or pharmacological androgen suppression. CRPC treatment is complicated by significant tumor heterogeneity, genetic diversity, escape mechanisms, medication resistance, recurrence, and restricted therapeutic options. A study which classified CRPC subtypes using ATAC-SEQ (Tang et al., 2022) revealed that AR-/low subtypes are primarily characterized by stem cell-like properties and Wnt pathway dependence. Additionally, transcriptomic signature analysis of 366 patients identified a stem cell-like subtype as the second most prevalent CRPC subtype, following the AR-dependent subtype. This indicated that prostate cancer stem cells (PCSCs) may play a crucial role in PCa developing diverse characteristics and resistance to therapies.

Within the prostate “niche,” (Liu et al., 2023; Borlongan et al., 2024) PCSCs exhibit self-renewal and pluripotency, which contribute to disease recurrence and treatment resistance (Yao and Zhang, 2022). Genetic or epigenetic alterations can convert normal prostate stem cells or prostate cells into PCSCs within the tumor microenvironment (TME) (Cheng et al., 2021). The genetic instability of cancer cells enables them to selectively adapt to different therapies through various mechanisms (Vlashi and Pajonk, 2015), including enhanced drug efflux, expression of anti-apoptotic genes, and active DNA repair (Kanwal et al., 2018; Li et al., 2018). Therefore, exploring PCSCs in the context of CRPC may reveal novel therapeutic strategies.

The physiological environment of PCSCs is regulated by several factors, including those from surrounding cells and stroma, which are in dynamic equilibrium. Cancer-associated fibroblasts (CAFs) play a crucial role in the TME by promoting PCSC stemness and maintaining a favorable environment. The interaction between CAFs and PCSCs can accelerate cancer progression (Loh and Ma, 2021). CAFs not only promote PCSC progression but also create an environment conducive to PCSC growth via abnormal signaling and remodeling of the surrounding tissue. Maintaining a favorable environment is crucial for cancer stem cell (CSC) survival and growth (Yoshida and Saya, 2021). PCSCs may also stimulate CAF growth and activation, thereby completing the communication cycle between these two cell subsets. Additionally, tumor treatment processes may enrich dysfunctional CAFs, which enhance PCSC stemness, further contributing to the CAF–PCSC crosstalk cycle (Huang T. X. et al., 2019).

In this review, we focus on the role of CAFs in maintaining PCSCs stemness within the TME. We discuss the potential of targeting CAFs, CAF-derived factors, and the extracellular matrix (ECM) to disrupt the coexistence of CAFs and PCSCs. Recent studies have demonstrated that interfering with these processes can deplete the self-renewal capacity and tumorigenic potential of PCSCs. Promising compounds are emerging from preclinical studies. Thus, strategies targeting the interaction between CAFs and PCSCs may provide new opportunities to combat PCa and help overcome therapeutic resistance.

## 2 PCSCs

### 2.1 Functional heterogeneity of PCSCs

Significant intra-tumor heterogeneity has been demonstrated throughout PCa progression (Gundem et al., 2015). Moreover, genomic sequencing of secondary metastases has revealed their subclonal heterogeneity (Hong et al., 2015). The origin and persistence of intra-tumor heterogeneity is closely linked to tumor stem cells (Batlle and Clevers, 2017). Tumorigenic cells with stem cell characteristics can initiate tumor development, sustain long-term growth (Mei et al., 2019), and self-renew to propagate tumors. These cells also give rise to non-tumorigenic cells, thereby generating heterogeneity that contributes to tumorigenesis, metastasis, and the development of drug resistance (Tsao et al., 2019). Thus, tumor stem cell subpopulations may lie at the apex of the tumor spectrum.

The evolution of PCa is marked by the dedifferentiation of cancer cells (Borges et al., 2015). Differentiated cells undergo transformation into stem cell-like or undifferentiated cells (Plaks et al., 2015). Additionally, when tumor cells gain the potential to become stem cells, “pluripotent” CSCs may regenerate into tumors. The functional differences within these tumors rely on the self-renewal capability of CSCs and their production of various tumor cells via asymmetric cell division (Kreso and Dick, 2014; Nassar and Blanpain, 2016; Batlle and Clevers, 2017). Histological remodeling of solid tumors includes different tumor cell types (Rahman et al., 2011). The self-renewal and metastatic abilities of PCSCs, together with their limited or reduced expression of androgen receptors (ARs), may contribute to the emergence of drug-resistant PCa and CRPC (Verma et al., 2023).

Genomic instability and epigenetic alterations (Nouruzi et al., 2022), including MYC activation, PTEN deletion, mutations in DNA repair genes (Halabi et al., 2016), and changes in the “stem cell ecological niche” (McGovern et al., 2009) contribute to tumor stem cell evolution. These cells adapt to the TME through mechanisms such as metabolic reprogramming (Yoshida, 2015), immunosuppressive phenotype development (Ma et al., 2018), rapid DNA repair (Skvortsov et al., 2015), ATP-binding cassette (ABC) transporter protein alterations (Begicevic and Falasca, 2017), and inflammatory or low-oxygen microenvironment adaptation (Yoshida and Saya, 2014).

PCSCs, derived from basal or luminal progenitor/stem cells, exhibit functional heterogeneity that influences the biological and clinical heterogeneity of Pca and its propensity for invasive behavior and treatment resistance (Zhang et al., 2016; Zhang et al., 2018b). PCSCs possess three main functional heterogeneities: tumor initiation, maintenance of cellular identity (self-renewal), and clonal evolution of different cells (differentiation programs) (Ward and Dirks, 2007). This ability of PCSCs becomes more prominent during disease progression and treatment, as evidenced by their differentiation into tumor cells with different phenotypes and functions in various TMEs (Gupta et al., 2019; Kuşoğlu and Biray Avcı, 2019). PCSCs are essentially androgen-independent and can grow in androgen-depleted environments. These cells proliferate and persistently differentiate into androgen-dependent and -independent cells in response to androgen deprivation therapy (ADT), leading to heterogeneous androgenic phenotypes in patients with CRPC (Gogola et al., 2023). This suggests that a genealogical switch occurs during PCa progression, which is consistent with tumor stem cell plasticity.

Collins et al. first identified CD44+/α2β1hi/CD133+ cells from PCa tumor tissues, which showed remarkable self-renewal and proliferative capacity (Collins et al., 2005). Since then, several studies have used a wide and heterogeneous range of markers to identify and isolate PCSCs (Harris and Kerr, 2017), including intracellular markers (e.g., ALDH), stem cell reprogramming factors, and transcriptional and epigenetic modulators (e.g., Oct3/4, Sox2, Klf4, Nanog, Myc, BMI1). *In vitro* tumorsphere assays and *in vivo* transplantation assays have been used to assess their properties (Civenni et al., 2019). These markers have been associated with self-renewal, stem formation, and promotion of CRPC (Kalantari et al., 2017; Verma et al., 2023).

### 2.2 PCSCs are key contributors to drug resistance

PCSCs exhibit intrinsic resistance to treatments (Lin et al., 2017), including endocrine therapy (Germann et al., 2012; Alumkal et al., 2020; Xu et al., 2022), chemotherapy (Domingo-Domenech et al., 2012; Lai et al., 2019; Li S. et al., 2020), radiotherapy (Chang et al., 2013; Tsao et al., 2019), and immunotherapy (Zhang et al., 2018a; Han et al., 2023). For example, ABCG2+ PCSCs are resistant to androgen receptor inhibitors (ARIs) due to increased androgen efflux (Huss et al., 2005). CD44^+^ (Wróbel et al., 2020), luminal-like (Sackmann Sala et al., 2017), and hypoxia-induced (O’Reilly et al., 2019) PCSCs exhibit inherent resistance to chemotherapy. Moreover, PCSCs can remain in a “resting” state for extended periods, highly express anti-apoptotic proteins (e.g., survivin and Bcl-2), and activate various signaling pathways, including those of Wnt, NF-κB, Notch, and Hedgehog. Therefore, PCSCs may develop resistance to conventional chemotherapeutic agents, such as docetaxel, that target rapidly dividing cells (Mimeault et al., 2008). Similar to many other CSCs, PCSCs are immunodeficient and lack the expression of many immunogenic molecules, rendering them insensitive to immunotherapy (Jeter et al., 2016; Miyashita et al., 2020).

An important mechanism underlying treatment resistance in PCa is lineage plasticity, which may be driven by PCSCs (Kushwaha et al., 2022). Anti-androgenic therapies, including ADT and novel endocrine therapies (Kregel et al., 2013), may enhance the capacity of PCSCs by facilitating the transition of AR+ PCa cells into CSC-like cells (Kanwal et al., 2018; Li et al., 2018), which re-differentiate into neuroendocrine-differentiated subtypes and other AR treatment-resistant subtypes (Verma et al., 2020). For example, AR gene suppression in PCa models (e.g., DU145 and LNCaP) increases self-renewal capacity and significantly upregulates relevant tumor stem cell markers, such as CD44, SOX2, and acetaldehyde dehydrogenase (ALDH) (Kregel et al., 2013). This suggests that PCa cells undergoing ADT can evade targeted therapy by altering their lineage plasticity and reprogramming into tumor stem cells. Moreover, extended enzalutamide therapy enhances CD133 and ALDH1A1 expression in LNCaP cells and modulates transcriptional signaling in PCa cells by increasing Nanog and OCT4 expression, leading to the development of stem cell-like characteristics (Verma et al., 2020). Mu et al. observed that CRPC cells developed resistance to enzalutamide by transitioning their phenotypic characteristics from AR-dependent luminal to AR-independent basal cells. This transition in lineage plasticity is induced by inactivation of tumor suppressor genes, including RB1 and TP53 (Mu et al., 2017). Furthermore, Zhang et al., discovered a substantial increase in β-catenin expression in enzalutamide-resistant cells. Activation of the Wnt/β-catenin pathway also leads to a considerable increase in the expression of stem cell markers. Furthermore, in a xenograft mouse model injected with patient LuCaP35CR cells, combining a β-catenin inhibitor, ICG001, with enzalutamide substantially decreased cancer cell proliferation, stem cell marker expression, and tumor development compared to treatment with enzalutamide alone (Zhang Z. et al., 2018).

### 2.3 Strategies and challenges in targeting PCSCs

Targeting various signaling pathways, including the Notch, Hedgehog, Wnt, and ABC transporter pathways, as well as the TME, may effectively control PCSCs (Li et al., 2017; Skvortsov et al., 2018). Several approaches have been devised to target these pathways, specifically using inhibitors or RNA silencing techniques (Koukourakis et al., 2023; Ramesh et al., 2023). Specifically, preclinical and clinical trials have assessed the efficacy of targeting the Notch pathway {RO4929097 [(Stein et al., 2012) and PF-03084014 (Du et al., 2018; Wang et al., 2020)], [Hedgehog pathway (sonidegib (Nanta et al., 2013), Gant-61 (Rimkus et al., 2016), and GDC-0449 (Tong et al., 2018)], Wnt pathway (LGK974 (Liu et al., 2013), OMP-54F28 (Le et al., 2015), Foxy-5 (Kelsey, 2017), and OMP-18R5 (Gurney et al., 2012)], and [ABC transporter protein pathways (cyclosporin A (Kawahara et al., 2015), dofequidar fumarate (Katayama et al., 2009), and vandetanib (Horti et al., 2009; Azad et al., 2014)]}. A recent study integrating single-cell RNA sequencing, spatial transcriptomics, and extensive ATAC sequencing identified a stemness subpopulation of cells labeled with SOX9highARlow expression that was significantly enriched after neoadjuvant ADT (Bian et al., 2024). Under antiandrogenic stress, PCA cells may transiently dedifferentiate into stem-like cells via plasticity-related pathways and redifferentiate into therapeutically resistant and invasive CRPCs. *In vitro*, *in vivo*, and clinical studies suggest that stem-like cells may be dependent on aberrant SOX9 expression. Inhibition of SOX9 expression ameliorates aggressive functional features associated with treatment resistance, including sphere formation and androgen-independent proliferation (Nouri et al., 2020). In another study using ATAC-seq, RNA-seq, and DNA sequencing (Tang et al., 2022), it was shown that the interaction of the AP-1 and YAP/TAZ pathways is critical for CRPC-stem cell-like (SCL) subtype-specific chromatin accessibility and gene expression. A small molecule inhibitor (vitexoporfin) employing the YAP/TAZ pathway, associated with a c-Fos/AP-1 inhibitor (T-5224), inhibited chromatin accessibility and gene expression.

Although these pathway inhibitors reduce tumor load and kill PCSCs, none are 100% effective owing to several limitations. The small number of tumor stem cells makes the identification of their specific proteins and markers challenging, complicating the accurate targeting of these cells. Most tumor stem cell markers discovered so far are shared with normal stem cells, and the identified signaling pathways are equally important for the self-renewal of both cell types. This similarity suggests that tumor stem cells may derive from normal stem cells; however, it limits the application of markers and signaling pathways for targeted therapies. Additionally, most receptors found in tumor stem cells are expression receptors rather than functional receptors, making them less suitable for therapeutic intervention. Furthermore, the heterogeneity of tumor stem cells further restricts targeted therapy, as multiple surface markers for various tumor stem cells can often be identified within the same tumor, complicating their identification and isolation (Peng et al., 2013; Lee et al., 2014). Another challenge for targeted therapy with tumor stem cells is the plasticity of tumor stem cells. Tumor cells are in a dynamic state of mutation and clonal evolution, and the plasticity of PCSCs in response to targeted therapy leads to the generation of different subclonal tumor cells. The high tumorigenicity of PCSCs also generates a small population of drug-resistant cells that eventually become the dominant cell population in the tumor tissue, leading to treatment failure (Mia et al., 2023; Quintero et al., 2023). Moreover, PCSCs engage in intricate interactions with cytokines, signaling molecules, the ECM, and other factors in the adjacent TME. Ecological niches protect PCSCs by maintaining and enhancing their stemness (Chaves et al., 2021; Runcie and Dallos, 2021; Han et al., 2023). The TME is usually stable and not easily disturbed by external factors. Therefore, tumor stem cells can be targeted more effectively by regulating non-tumor cells and their derived factors in the TME.

### 2.4 PCSCs and the TME

In 1889, Stephen Paget proposed the “seed and soil” hypothesis, which states that tumor cells are comparable to “seeds” in the “soil” of the TME, wherein the two engage in a bidirectional and dynamic balance that determines tumor growth (Paget, 1989). Normal stem cells exist in a “stem cell envelope,” comprising different cell types and extracellular matrices (Borovski et al., 2011), which maintains their stemness. Recent data suggest that CSCs depend on a similar ecological niche, called the “CSCs ecosystem,” comprising CAFs, immune cells, and the ECM, among others. (Pedro et al., 2016; Huang et al., 2018; Brown and James, 2021). During the early stages of tumorigenesis, PCSCs are regulated by multiple signaling pathways and the TME, which continues to undergo adaptive evolution (Chiarugi et al., 2014), including ECM remodeling, which involves the secretion of many growth factors and matrix-degrading enzymes; leading to the formation of a hypoxic, inflammatory, and immune-suppressive microenvironment. This evolution serves to maintain the ecological niche of PCSCs (Jaworska et al., 2015), by facilitating the maintenance and regulation of undifferentiated PCSCs, as well as the regulation of self-renewal and differentiation of PCSCs (Williams et al., 2013). The PCSC ecological niche generates CSC properties by inducing tumor cell dedifferentiation. Analysis of gene expression in prostate α2β1hi/CD133+ cells revealed an increase in the activity of ECM integrin signaling, which is associated with CSCs. Therefore, PCSCs may exhibit distinct responses to, or undergo changes, in specific environments. Specifically, PCSCs upregulate the expression of integrin αv and laminin chains (α1, α5, γ1) (Birnie et al., 2008). Knockout and complementation experiments support the idea that secreted protein acidic and cysteine rich (SPARC) protein (i.e., osteonectin), a matrix glycoprotein that regulates tissue repair and remodeling of the ECM, is a major factor regulating CSC and non-CSC cooperation (Mateo et al., 2014).

CAFs are crucial components of the TME that help regulate the growth of various malignancies, including PCa. Specifically, CAFs may enhance gland development in PCSCs (Giannoni et al., 2010). Moreover, PCa cells undergoing epithelial-mesenchymal transition (EMT) driven by CAFs exhibit upregulation of CSC-associated markers, which are related to tumor invasion and metastasis. Thus, selective targeting CAFs may regulate the pluripotency of PCSCs, thereby facilitating the development of novel treatment strategies (Giannoni et al., 2010; Giannoni et al., 2011).

## 3 Crucial function of CAFs in PCa development

### 3.1 Functional heterogeneity of prostate CAFs

The process of prostate epithelial tumor transformation is dependent on its surroundings and relies on the interaction between cancer and stromal cells (Torrealba et al., 2017). Tumor-associated stromal cells undergo synergistic changes during the early stages of PCa development. Prostate stromal cells, such as fibroblasts, play a major role in this process. Additionally, stromal cells in precancerous lesions and PCa undergo changes similar to those observed during wound healing. The abundance of this particular class of stromal cells, characterized by the CAF phenotype, increases as the tumor progresses to a more advanced stage (Bonollo et al., 2020). During the evolution of CAFs, the prostatic mesenchyme undergoes phenotypic changes (Levesque and Nelson, 2018), including the transformation of fibroblasts into “myofibroblast-like” cells, ECM deposition, neovascularization, and immune cell infiltration, resembling the inflammatory process of wound healing (Belhabib et al., 2021). This involves the gradual replacement of well-differentiated stromal smooth muscle cells (Sahai et al., 2020). Specifically, fibroblasts participate in ECM formation by secreting type I and III collagens and upregulating CAF-specific marker expression such as waveform protein, fibroblast activation protein (FAP), α-smooth muscle actin (α-SMA), and platelet-derived growth factor receptors (PDGF-R), while downregulating junction protein expression and decreasing the synthesis of proteases that regulate the ECM and ECM remodeling (Gandellini et al., 2015).

CAFs can be recruited by tumors from other sources or originate from fibroblast transformation (LeBleu and Kalluri, 2018). For example, epithelial cells that have undergone EMT are associated with CAF activation, and mesenchymal stem cells (MSCs), fibroblasts, and endothelial cells may also be recruited and differentiated into CAFs (Shiga et al., 2015; Shi et al., 2017). CSCs may also be a key source of CAFs in the TME; mammary CSCs activate Hedgehog signaling via paracrine transmission to regulate CAFs. In response to Hedgehog activation, CAFs secrete activin A insulin-like growth factor 1 and leukemia inhibitory factor (LIF), which further supports CSC growth and proliferation (Valenti et al., 2017). Nair et al. (2017) used pluripotent stem cells to generate CSC-like cells, which form a heterogeneous population surrounded by myofibroblast-like cells. Treating these cells with transforming growth factor (TGF)-β1 induces their differentiation into CAFs expressing FSP1, vimentin, COL1α1, and CXCL12. In some cancers, CSCs acquire a myofibroblast-like phenotype via EMT (Petersen et al., 2003). EMT-derived CAFs carry multiple cancer cell-like mutations, whereas CAFs of other origins undergo additional genetic and/or epigenetic changes to become malignant. Hence, EMT may represent a trans-differentiation regimen that allows CSCs and precursor cells to produce CAFs in certain tissues, promoting tumor growth and metastasis (Huang et al., 2015).

CAFs exhibit significant heterogeneity (Ishii et al., 2016), as evidenced by their ability to transform phenotypically different CAFs into one another (Sahai et al., 2020). Inflammatory fibroblasts (iCAFs) and myofibroblasts (myCAFs) have immunomodulatory and matrix-producing contractile phenotypes that are functionally distinct and often mutually exclusive (Öhlund et al., 2017). Interleukin (IL)-1 triggers JAK/STAT activation, leading to iCAF production, whereas TGF-β counteracts this mechanism to facilitate myCAF conversion (Chan et al., 2022; Deng et al., 2022). Recent studies have identified a new CAF subtype, the antigen-presenting or immunomodulatory class of CAFs (apCAFs), which express MHC class II-related genes and stimulate T-cell receptor attachment (Biffi and Tuveson, 2021). The interchange between CAF isoforms contributes to tumor growth and therapeutic resistance.

Wang et al. (2023) reported changes in the fibroblast composition of CRPC, where myCAFs correlated with antiandrogen resistance. MyCAFs under androgen therapy promote PCa cell survival and growth. Mechanistically, antiandrogen treatment is a prerequisite for TGF-β signaling activation in fibroblasts, which induces cellular reprogramming of CAFs in PCa. Inhibition of AR signaling with enzalutamide sensitizes CAFs to TGF-β1 treatment, indicating that AR functions as a checkpoint to suppress TGF-β signaling. Blocking AR activity increases TGF-βR1 production and activation, stimulating SOX4 expression. SOX4 interacts with SWI/SNF to modify chromatin accessibility, causing the transformation of PDGFRα+ iCAFs into myofibroblast-like CRPC-CAFs (Mittal and Roberts, 2020; Li and Mu, 2023).

### 3.2 CAFs contribute to PCa development

CAFs show a significant capacity for facilitating the development and advancement of drug resistance in tumors. For example, PCa cells grown with CAFs in a coculture system exhibit heightened resistance to enzalutamide and bicalutamide (Chandra Jena et al., 2021). Therefore, targeting CAFs and their downstream effectors and signaling pathways may enhance their sensitivity to anticancer therapy.

#### 3.2.1 Paracrine signaling by CAFs promotes PCa progression and treatment resistance

CAFs enhance the growth of PCa cells by secreting soluble factors such as interleukins, interferons (IFNs), and tumor necrosis factor (TNF). Among these factors, TGF-β plays a crucial role in activating CAFs and contributes to the genesis and maintenance of the CAF phenotype (Massagué et al., 2000). TGF-β may regulate CAF function via the cross-regulation of several signaling pathways, including PI3K/AKT, CXCR4/CXCL12, and MAPK/ERK (Kiskowski et al., 2011; Avgustinova et al., 2016). LNCaP cells overexpressing TGF-β1 induce prostate MSCs to differentiate into a reactive myofibroblast phenotype associated with CAFs (Verona et al., 2007). Meanwhile, TGF-β derived from CAFs promotes tumor progression by increasing TGF-β1 expression and secretion in conditioned media, contributing to PCa cell proliferation and migration (Sun et al., 2019).

Furthermore, CAFs secrete fibroblast growth factor (FGF) with tumor growth-stimulating effects (Kwabi Addo et al., 2004). The FGF receptor (FGFR) signaling pathway activates many downstream cascades, including the RAS/MAPK, PI3K/AKT/mTOR, and JAK/STAT signaling pathways (Ferguson et al., 2021). FGFR interacts directly with cell adhesion molecules and ECM proteins to promote cancer cell invasion and migration, whereas interactions with other receptor tyrosine kinases (RTKs) regulate cancer angiogenesis, treatment resistance, and metastatic potential (Wang C. et al., 2019). Thus, the CAF-mediated ectopic FGF signaling axis disrupts tissue homeostasis and induces PCa.

Bidirectional crosstalk between tumor cells and CAFs enhances the ability of fibroblasts to secrete various pro-tumor chemokines, including CXCL12/CXCR4 axis (Mishra et al., 2011), CCL2, CCL5, CCL7, CXCL8, and CXCL14 (Toledo et al., 2022). Chemokines and their receptors play functional roles in PCa development, particularly in bone metastasis (Sharma et al., 2022). Specifically, the CXCL12/CXCR4 axis enhances cancer cell invasiveness and migratory capacity by increasing the expression of EMT markers, whereas CXCR4 knockdown significantly reduces the migration and invasion of PCa cells into osteoblasts (Huang Z. et al., 2019). Furthermore, upregulation of CXCL12γ induces the development of tumor stem and neuroendocrine phenotypes in PCa cells by activating PKCα/NF-κB signaling through CXCR4 (Jung et al., 2018).

Additionally, CAF-derived IL-6 may regulate cell stemness by promoting STAT3 phosphorylation, thereby contributing to enzalutamide resistance (Culig and Puhr, 2018). IL-6/STAT3 tumor stemness may be associated with elevated reactive oxygen species (ROS) levels (Qu et al., 2013). IL-6 may also modulate AR function by enhancing AR transactivation via the STAT3 or MAPK pathway (Yang et al., 2003). Furthermore, activation of the STAT3/NFκB pathway by CAF-secreted IL-6 induces CXCR7 expression in tumor cells, which has been associated with drug resistance (Qiao et al., 2018). CXCR7 acts as a CXCL12 scavenger receptor and is highly expressed in ENZ-resistant mouse models. In CRPC, AR suppresses CXCR7 by directly interacting with an enhancer located 110 kb downstream of the gene (Li et al., 2019; Rafiei et al., 2019). Hence, the inhibition of AR activity via enzalutamide releases CXCR7, promoting CRPC cell progression by increasing their ability to resist apoptosis, proliferate rapidly, repair DNA, and initiate angiogenesis (Luo et al., 2018).

CAFs develop resistance to enzalutamide or ADT via the binding of secreted neuregulin 1 (NRG1) to human epidermal growth factor receptor 3 (HER3) in cancer cells. Blocking the NRG1/HER3 axis effectively inhibits tumor cell resistance to antiandrogen therapy (Zhang et al., 2020). NRG enhances the growth of CRPC CWR-R1 and 22RV1 cells and activates AR (Gregory et al., 2005); this effect is more pronounced in cultures with denuded androgen levels.

CAFs drive chemotherapy resistance, whereas stimulation of the traditional Wnt pathway induces EMT in PCa cells by increasing the secretion of WNT16B—a source of CAFs—via NF-κB signaling (Sun et al., 2012). Additionally, CAFs enhance chemoresistance by suppressing P53 activity; the conditioned media of CAFs decreases P53 expression in PCa cells, facilitating cell survival (Cheteh et al., 2017). Bone-derived CAFs secrete CXCL12, and activated CXCL12/CXCR4 signaling promotes docetaxel resistance in PC3 cells and bone metastasis, which can be reversed by blocking the CXCR4/CXCL12 axis (Domanska et al., 2012).

PCa is characterized as a “cold tumor” due to the lack of tumor T-cell infiltration, leading to resistance to immune checkpoint inhibitors (ICIs). CAFs are crucial for regulating the antitumor activity of tumor-infiltrating lymphocytes (TILs) (Harper and Sainson, 2014; Liu et al., 2019). CAFs direct immune cell recruitment to the TME by secreting soluble factors and altering immune cell permeability. CAFs also enhance the expression of immunological checkpoint molecules and facilitate ECM remodeling, indirectly influencing recruitment and function (Vickman et al., 2020). PCa induces tumor immune evasion by activating and recruiting CAFs via TGF-β secretion. This process depletes immunosuppressive CD8^+^ Tregs and enhances tumor development, while increasing ICI resistance (Davidsson et al., 2013).

#### 3.2.2 Role of CAFs in ECM remodeling

In addition to their paracrine effects, CAFs accelerate ECM deposition and turnover (Clark et al., 2013) by increasing the production of ECM-associated substances (collagen, tendonogenic protein-C, and fibronectin) (Josefsson et al., 2011). ECM deposition by reactive stromal cells stimulates PCa cell growth, and ECM derived from CAFs specifically enhances LNCaP cell proliferation (Palumbo et al., 2012). In spheroidal cocultures PCa cells and CAFs, an accumulation of basement membrane linkage proteins, heparan sulfate proteoglycans, and collagen XVIII has been observed (Ojalill et al., 2020). This increased ECM circulation can produce cytokines, including growth differentiation factor 15 (GDF15) and TGF-β, which enhance tumor cell proliferation and invasion (Scott et al., 2019). Moreover, the overexpression of matrix metalloproteinases, (MMP)-1, -2, -7, -9, and -14, suggests that prostate CAFs induce ECM remodeling via MMP secretion, which enhances PCa cell invasiveness (Gong et al., 2014).

CAFs can modify the physical characteristics of the TME, thereby affecting treatment efficacy. A robust association between CAF-induced ECM remodeling and the development of drug resistance in tumors has been described (Feng et al., 2022). CAFs influence cell adhesion, proliferation, and fibrosis in the tumor stroma by generating large amounts of collagen, fibronectin, and other stromal components, thereby increasing the stiffness and pressure of the TME (Guo et al., 2020). This creates a hypoxic and metabolically stressful environment, increases the expression of anti-apoptotic proteins and drug-resistant signaling pathways (Senthebane et al., 2017), and effectively reduces drug penetration and efficacy by establishing a barrier between cancer cells and therapeutic drugs (Henke et al., 2019). Analysis of RNA-seq data from The Cancer Genome Atlas (TCGA) revealed that dysregulated ECM-related genes in CAFs promote TGF-β signaling and ICI resistance. This suggests that CAFs may facilitate immune evasion and immunotherapeutic resistance via EMC-related genes through adaptive mechanisms (Ghahremanifard et al., 2020). Thus, targeting the ECM remodeling capabilities of CAFs could enhance the effectiveness of cancer therapies by improving drug delivery and reducing resistance.

## 4 Role of CAFs in maintaining PCSC stemness

### 4.1 CAFs in coculture systems enhance PCSC properties

**FIGURE 1:**
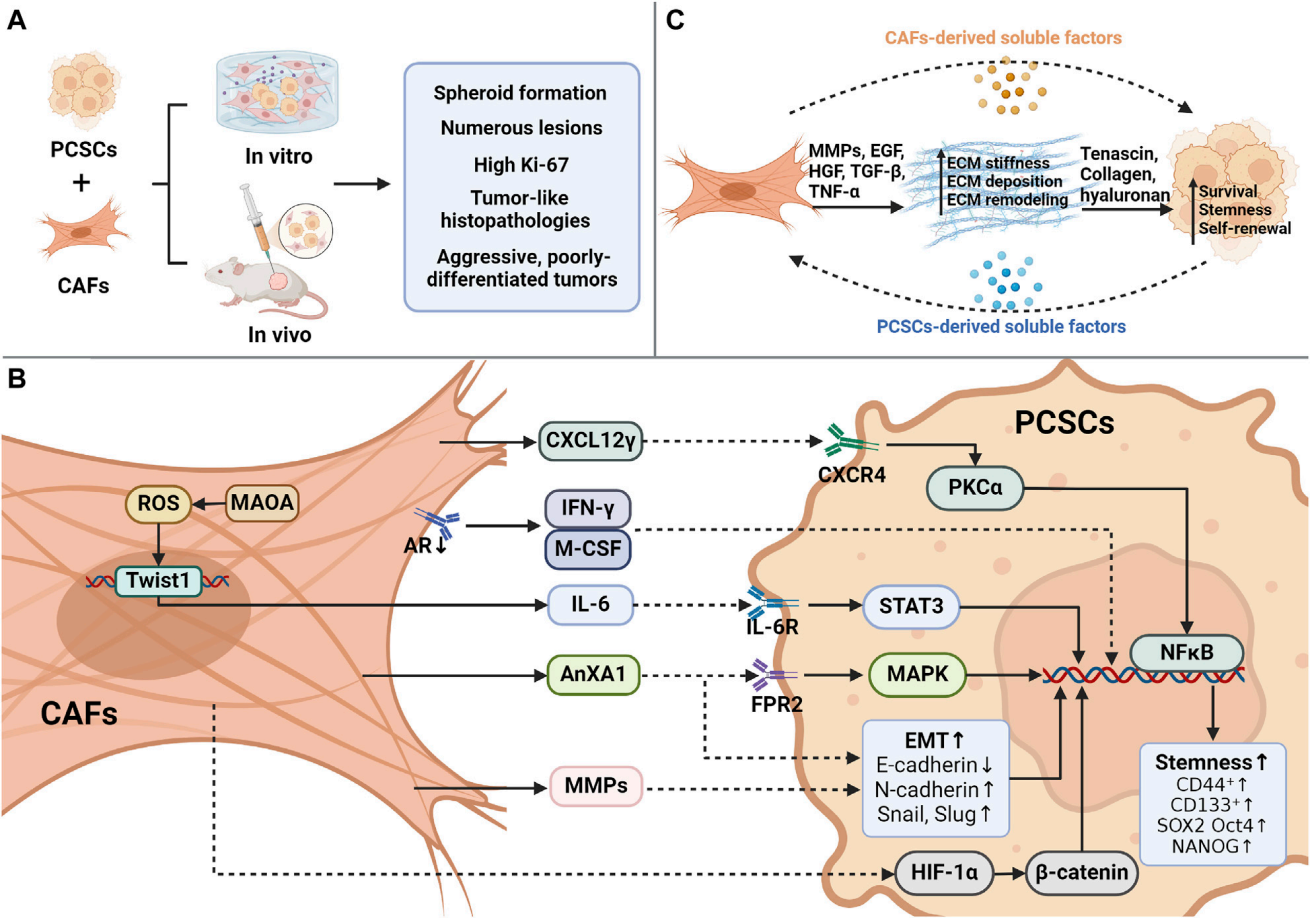
Interaction between CAFs and CSCs. **(A)** During the coculture of cancer-associated fibroblasts (CAFs) and prostate cancer stem cells (PCSCs), CAFs promote sphere formation in contrast to normal fibroblasts and develop more lesions, higher proliferation indices, and tumor-like histological features *in vivo*. **(B)** Overview of the mechanism of CAF-mediated paracrine action in promoting PCSC stemness. CAFs can orchestrate several processes, including the secretion of CXCL12γ, which binds to CXCR4 on the membrane of PCSCs, activates PKCα/NFκB, and promotes stemness. IFN-γ/M-CSF secretion induces PCSC marker expression after androgen receptor (AR) inhibition by CAFs. The presence of monoamine oxidase A (MAOA) in CAFs triggers the transcription and secretion of IL-6 in a ROS-dependent manner, activating IL-6/STAT3 signaling to promote PCSC stemness and CD44 expression. Induction of EMT by CAF-derived AnxA1 generates CSC-like cells from PCa epithelial cells. CAF-secreted MMPs trigger an increase in EMT, CD44^+^/CD24^-^ ratio, and CD133^+^ expression in tumor cells. CAFs stimulate PCSCs through the HIF-1α/β-catenin-dependent signaling pathway that drives PCSCs to undergo EMT. **(C)** CAFs regulate the ecological niche of PCSCs through cellular matrix remodeling. Created using Biorender.com.

Conditioned media from CAF cultures or CAF and tumor cell cocultures can increase the stemness of tumor cells. This is achieved by inducing the expression of stemness markers, such as SOX2, OCT4, Nanog, CD44, and CD133. Additionally, CAFs enhance sphere formation *in vitro* and stimulate CSC self-renewal and proliferation in lung (Su et al., 2018), prostate (Liao et al., 2017), breast (Tsuyada et al., 2012), colorectal (Ren et al., 2018), and gastric (Hasegawa et al., 2014) cancers. In a PCa and CAF coculture system, CAFs acquire a phenotype that maintains cancer stemness when the corresponding stimuli from tumor cells or the microenvironment activate specific signaling pathways. The kinetics of CAF differentiation toward a luminal cell phenotype are better exhibited than those of basal cell types relative to other fibroblasts. In a coculture system of CAFs and CSCs isolated from a Pten-deficient mouse model of PCa, CAFs promoted sphere formation compared to normal prostate fibroblasts. *In vivo* transplantation tumor experiments further demonstrated that prostate glandular structures formed by mixing CSCs with CAFs contained more lesions, higher proliferation index, and tumor-like histopathology. Furthermore, CAFs facilitate a two-fold augmentation in sphere formation compared to urogenital sinus mesenchymal cells and normal prostate fibroblasts (Liao et al., 2010a). Moreover, when CSCs were mixed with CAFs or NPFs and examined in live kidney transplants, grafts formed from CAFs exhibited a higher proliferation index and more lesions with more complex morphology than NPFs (Liao et al., 2010b). Adisetiyo et al. (2014) found that CRPC-derived CAFs (CRPCAF) induced aggressive, poorly differentiated tumors when combined with CRPC-derived CSCs (CRPCSCs). Compared with hormone-sensitive PCa-derived CAFs (ADPCAFs), CRPCAFs in NOD/SCID mice formed more glandular structures and supported the development of aggressive, poorly differentiated tumors, as evidenced by a higher Ki67 index. Hence, the paracrine factors released by CRPCAFs specifically promote the stemness and tumorigenic features of the relevant CSCs (Figure 1A).

### 4.2 Paracrine signaling by CAFs enhances PCSC characteristics

CAFs secrete many soluble molecules, including IL-6, IL-8, CXCL1, CXCL12, hepatocyte growth factor (HGF), and TGF-β, which regulate CSC stemness via paracrine signaling (Xiong et al., 2018). Activation of downstream CXCR4 signaling by CAF-derived CXCL12 promotes EMT and contributes to tumor stem cell activity (Rajasekhar et al., 2011). CXCL12γ promotes tumor development in CRPC, whereas many CD133+/CD44+ CSC-like cells are present in CXCL12γ-overexpressing tumors. This leads to a PCSC phenotype by activating the PKCα/NF-κB pathway via CXCR4. Consequently, the number of metastatic tumor cells in soft and bone tissues increases (Jung et al., 2018). However, inhibition of PCSC sphere formation by blocking the CXCL12/CXCR4 axis with a CXCR4 receptor antagonist (AMD3100) or an antibody restores the chemosensitivity of PCSCs (Dubrovska et al., 2012).

AnxA1 is derived from CAFs and increases the CSC-like characteristics of progenitor cells. This was demonstrated *in vitro* by the formation of more intricate spheroids. *In vivo*, AnxA1 leads to the development of large, histologically complex glandular structures and increases p63 expression. Thus, AnxA1 may enhance the function of PCSCs via two separate but mutually supportive mechanisms: 1) pERK1/2 activation leads to the induction of a dedifferentiation process in PCa epithelial cells, comparable to the intermediate stage of EMT, and 2) the growth and specialization of PCSCs are facilitated (Geary et al., 2014). Monoamine oxidase A (MAOA) in CAFs supports the development of an inflammatory and pro-tumorigenic ECM via paracrine IL-6/STAT3 signaling. MAOA stimulates the transcription and secretion of IL-6 in a ROS-dependent manner, leading to the induction of oxidative stress. Subsequently, the IL-6/STAT3 signaling pathway is activated, promoting the maintenance of stemness within the cytosol and CD44 expression (Li J. et al., 2020). However, IL-6 may also trigger cellular oxidative stress via several mechanisms (Ene et al., 2022). Therefore, MAOA-mediated modulation of ROS/IL-6 interactions may create a harmful cycle that promotes the transformation of naïve stroma into a phenotype that supports tumor growth. In pathological tissues, CAFs drive the development of PCSCs by stimulating the HIF-1α/β-catenin-dependent signaling pathway, initiating an EMT program. With the acquisition of EMT traits, PCSCs dynamically acquire higher migratory capacity (Luo et al., 2013). The presence of active and functional ARs in CAFs is crucial for regulating the stemness of PCSCs. When the expression of ARs is suppressed in CAFs via an AR antisense oligonucleotide, a significant increase in the formation of spherical structures and the expression of CSC markers in LNCaP cells occurs in coculture systems. Moreover, AR deletion upregulates interferon-gamma (IFN-γ) and macrophage colony-stimulating factor (M-CSF), resulting in the acquisition of CSC-like characteristics by PCa cells (Liao et al., 2010b). Paracrine interactions between CAFs and cancer cells result in EMT-driven enhancement of CSC characteristics linked to invasiveness and metastasis. PCa cells produce IL-6, which influences CAF activation. Subsequently, CAFs secrete MMP2 and MMP9, which trigger ROS production in cancer cells via the RAC1B/COX-2 pathway. Elevated ROS levels cause NF-κB translocation and HIF-1 activation, leading to an increase in the CD44+/CD24-ratio and CD133 expression. Ultimately, these processes enhance the clonogenic, self-renewal, and proliferative capabilities of PCa cells (Giannoni et al., 2010; Giannoni et al., 2011) (Figure 1B; Table 1).

**TABLE 1:** Effects of CAF-derived factors on prostate cancer stemness.

Factor	Function	Stemness pathway involved	Reference
CXCL12γ	Promotes cancer stemness and neuroendocrine phenotypes	CXCL12γ/CXCR4, PKCα/NFκB	Jung et al. (2018)
AnxA1	Maintains EMT phenotype and stem cell properties	pERK1/2, TGF-β1/TGFβRII	Geary et al. (2014)
MAOA	Induces CSC marker expression	Twist1/IL-6/STAT3	Li et al. (2020a)
—	Enriches CSCs and promotes cancer cell proliferation and invasion	HIF-1α/β-catenin	Luo et al. (2013)
IFN-γ/M-CSF	Induces CSC marker expression		Liao et al. (2017)
MMPs (MMP-2, MMP-9)	PCSC secretes IL-6 to cultivate pro-tumor CAF, which promotes stemness		Giannoni et al. (2010)
MMPs	Maintains EMT phenotype and stem cell properties	RAC1B/COX-2, NF-κB/HIF-1	Giannoni et al. (2011)

CAFs, Cancer-associated fibroblasts; CXCL12γ, chemokine (C-X-C motif) ligand 12γ; CXCR4, C-X-C chemokine receptor type 4; PKCα, Protein kinase C α; NFκB, nuclear factor kappa-B; AnxA1, Annexin A1; EMT, epithelial-mesenchymal transition; pERK1, proline extensin-like receptor kinase 1; TGF-β1, transforming growth factor-β1; TGFβRII, transforming growth factor-β type II receptor; MAOA, monoamine oxidase A; CSC, cancer stem cell; IL-6, interleukin-6; STAT3, signal transducer and activator of transcription 3; HIF-1α, hypoxia-inducible factor-1α; IFN-γ, interferon-γ; M-CSF, macrophage-stimulating factor; MMPs, matrix metalloproteinases; PCSC, prostate cancer stem cell; Rac1b, RAS-related C3 botulinum toxin substrate 1b; COX-2, cyclooxygenase-2; HIF-1, hypoxia-inducible factor 1.

### 4.3 CAFs mediate cancer stemness via ECM remodeling

PCSCs form their own “niches” by recruiting and activating specialized cells anchored to a specific local ecological niche via ECM receptors. This not only allows cells in the niche to maintain stemness by secreting soluble factors that act directly on stem cells (Li and Xie, 2005) but also maintains the polarity of the stem cells, orienting them to undergo mitosis, spindle division, and asymmetric cell division. This stromal environment, enriched with oncogenic cells, protects CSCs from differentiation and limits their proportion and growth space, thereby contributing to the maintenance of stemness (Fessler et al., 2013). CSC markers and CSC graft tumor growth are inhibited when CSCs lose their ecological niche (Valent et al., 2012).

As major components of the CSC ecological niche (Cully, 2018; Prager et al., 2019), CAFs provide a mechanically supportive niche for newly formed CSCs. CAFs secrete growth factors, including EGF, HGF, and TGF-β, as well as proinflammatory cytokines (e.g., TNF-α) to facilitate niche creation (De Veirman et al., 2014; Kfoury and Scadden, 2015). Activated CAFs participate in dynamic ECM homeostasis via ECM remodeling (Josefsson et al., 2011) and the production of ECM-degrading enzymes to aid the communication and transport of inflammatory cells (Kalluri, 2016; Ojalill et al., 2020). In PCa, CAFs overproduce fibronectin and collagen, leading to morphological changes and increased ECM stiffness (Bonollo et al., 2020). Softer (Ng et al., 2021) and stiffer (Tao et al., 2021) matrices can trigger tumor stem cell stemness. Collagen deposited by CAFs serves as a mechanical signal that promotes stem cell markers and sphere formation (Cazet et al., 2018). Additionally, hyaluronic acid secreted by CAFs is a major component that promotes self-renewal and EMT in CSCs (Chanmee et al., 2015). The ECM serves as a mechanical barrier that blocks drug action, contributing to drug resistance in tumor stem cells (Casazza et al., 2014) (Figure 1C).

## 5 Potential new therapeutic strategies for targeting CAFs to regulate PCSCs

CAF has been identified as a promising target for prostate cancer treatment due to several reasons: i) CAFs have a higher genetic stability compared to tumor cells, which reduces the likelihood of developing drug resistance; ii) CAFs primarily contribute to the remodeling of the extracellular environment, creating a physical barrier that hinders the effectiveness of anticancer drugs; and iii) CAFs play a role in creating an immune-suppressing environment or maintaining a microenvironment that supports the survival of cancer stem cells (Clark et al., 2022). A large number of preclinical studies have been reported, however only a few clinical trials have been conducted using drugs or strategies specifically targeting CAFs in cancer patients. This is due to a lack of understanding of the key processes involved in CAFs biology and the complexity of this heterogeneous group of markers, which hinders the translation of CAF-focused strategies into clinical practice. The exploration of the efficacy of targeting CAFs as a potential therapeutic option has progressed since the first clinical trials using a monoclonal antibody to FAP+ CAFs in metastatic colon cancer (Welt et al., 1994). Therapeutic strategies continue to evolve to counteract the complexities associated with the molecular, functional, and spatial heterogeneity of CAFs in various cancers (Bejarano et al., 2021; Chen et al., 2021). A number of different strategies have been explored to target CAFs and their activity in the TME, including direct depletion of the CAFs population, inhibition of downstream CAFs signaling pathways and CAF-involved extracellular matrix remodeling.

### 5.1 Depletion of CAFs for pro-tumor phenotypes

As mentioned previously, the lack of specific markers for CAFs poses a significant obstacle to depletion strategies. Numerous studies have now employed various means to achieve direct depletion of CAFs, including antibody-directed targeting of cells expressing markers for CAFs or utilizing these markers as targets for other drug delivery. Several recent studies have shown some promise for this approach. Androgens enable PCa cells to recruit CAFs and mediate PCa cell migration and invasion by assembling the AR/filamentous in A (FlnA) complex in CAFs, thereby triggering ECM remodeling. A peptide (Rh-2025u) that interferes with the AR/FlnA complex was used to disrupt the cellular network around PCa-like organs, reduce biochemical changes in the ECM structure, and impair CAF migration to PCa cells (Di Donato et al., 2021). Increased yes-associated protein 1 (YAP1) levels are present in the surrounding tumor tissue in clinical PCa samples with a high Gleason grade and are positively associated with metastasis and poor prognosis (Gamze et al., 2015). Elevated YAP1 expression triggers differentiation of prostate fibroblasts into CAFs via the YAP1/TEAD1 protein complex. This complex regulates cytoskeletal proteins and actin downstream of SRC transcription, thereby contributing to the CAF phenotype (Calvo et al., 2013). Reducing YAP1 activity in CAFs using siYAP1 and the inhibitor verteporfin (VP) significantly inhibits the proliferative capacity of CAFs (Shen et al., 2020).

A nanoparticle siRNA delivery system targeting CAFs for reprogramming has shown effectiveness in preclinical settings. This system was developed using a cell-penetrating peptide (CPP) to specifically target CAFs. Additionally, a nanosystem comprising PNP/siCXCL12/mAb was designed to deliver CXCL12-silencing siRNA to CAFs. This was achieved by loading the system with siRNA and adsorbing it with an anti-FAP-α monoclonal antibody on its surface. Targeting FAP-α facilitates specific delivery of siRNA to CAFs, resulting in the downregulation of CXCL12 expression. Consequently, maintenance of the pro-tumorigenic CAF phenotype is inhibited (Lang et al., 2019). Moreover, TGF-β signaling contributes to CAF activation and enhances PCSC stemness (Jiao et al., 2020). GKT137831, an NOX1/4 inhibitor, impedes TGF-β1-induced ROS production mediated by CAFs, leading to a decrease in α-SMA and vimentin expression (Sampson et al., 2018). A phase I human trial (NCT03089203) used CAR - T cells with a dominant-negative TGF-β receptor for the treatment of metastatic denervation-resistant prostate cancer (mCRPC) (Narayan et al., 2022). Serum PSA levels decreased by 36% in patients treated with CART-PSMA-TGFβRDN cells, indicating tumor regression. Thus, the clinical application of TGF-β-resistant CAR-T cells is feasible and generally safe.

Numerous extracts have demonstrated efficacy in specifically targeting CAFs. For instance, silymarin directly suppresses CAF-like transformation of naïve fibroblasts and indirectly hinders the ability of PCa cells to secrete TGF-β2, which for induces CAFs. Specifically, silymarin significantly decreased the production of TGF-β2 and biomarkers associated with CAFs in tumors (Ting et al., 2015). Moreover, cinnamaldehyde (CA) induces cell cycle arrest and apoptosis in prostate CAFs via endogenous pathways, including induction of endolipid membrane ectopy, decreased mitochondrial membrane potential, elevated intracellular ROS and calcium levels, and activation of the caspase family (Han et al., 2020). Curcumin-induced ROS upregulation triggers endoplasmic reticulum (ER) stress in prostate CAFs via the PERK/eIF2α/ATF4 axis, thereby inhibiting prostate CAFs via apoptosis and G2–M phase cell cycle arrest (Zeng et al., 2020). Additionally, curcumin inhibits CAF-induced PCa invasion and mesenchymal transition by blocking the MAOA/mTOR/HIF-1α axis, reducing ROS generation, and suppressing the expression of CXCR4 and IL-6 receptors (Du et al., 2015). In summary, by interfering with the AR/FlnA complex, targeting several extracts, and inhibiting the activity of key factors, such as YAP1, CXCL12, and TGF-β, the pro-tumor function and ability to maintain the CAF tumor can be effectively impaired, providing a new approach for PCa treatment (Table 2).

**TABLE 2:** Candidate strategies for targeting CAF–PCSC interactions.

Function	Method or drug candidate	Target	Mechanism	Reference
Targeting CAFs	Rh-2025u	AR/FlnA complex	Abolishes the androgen-dependent migration and invasiveness of CAFs	Di Donato et al. (2021)
	siYAP1 and the inhibitor verteporfin (VP)	YAP1/TEAD1 protein complex	Disruption of the YAP1/TEAD1 protein complex reverses SRC-mediated conversion of NFs to CAFs by cytoskeletal proteins	Calvo et al. (2013), Shen et al. (2020)
	PNP/siCXCL12/mAb	CXCL12	Targets FAP-α in CAFs and inhibits CXCL12 gene expression, inhibiting CAFs	Lang et al. (2019)
	GKT137831	Nox4/TGF-β1	Abrogates TGF-β1-driven fibroblast activation via NOX4 inhibition	Sampson et al. (2018)
	Silibinin	TGF-β2	Direct or indirect inhibition of CAFs	Ting et al. (2015)
	Cinnamaldehyde	GSH	Interferes with mitochondrial function related to glutathione, leading to induction of endogenous apoptosis in prostate CAFs	Han et al. (2020)
	Curcumin	ROS/(PERK-eIF2 α-ATF4 axis)	Induces programmed cell death in CAFs by generating ROS and subsequent activation of endoplasmic reticulum stress	Zeng et al. (2020)
		MAOA/mTOR/HIF-1α signaling	Suppresses CAF-induced invasion and EMT, reduces ROS generation and the expression of CXCR4 and IL-6 receptors in PCa cells by blocking the MAOA/mTOR/HIF-1α signaling pathway	Du et al. (2015)
Target CAF-derived factors	IL-6-neutralizing antibody, STAT3 siRNA, ilomastat	IL-6/IL-6R/STAT3/MMPs	Attenuates the expression of CSC markers, CSC-like properties *in vitro* and *in vivo*	Giannoni et al. (2011)
	AMD3100	CXCL12/CXCR4	Suppresses CSC phenotype induced by the CXCL12/CXCR4 pathway	Jung et al. (2018)
	Clorgyline	MAOA/IL-6/STAT3	Inhibits downstream STAT3 signaling-mediated cell stemness and CD44 expression	Li et al. (2020a)
Target ECM	PXS-S2a	LOXL2	Realigns the ECM structure generated by CAFs to that of healthy fibroblasts	Nguyen et al. (2019)
	Cleavable amphiphilic peptide (CAP)	FAP-α	Breaks the matrix barrier	Ji et al. (2016)
	AF307	PDGFRα	Alters stromal organization of CAF	Borges et al. (2015)
	Antiarrhythmic drugs (amiodarone, verapamil, nifedipine, flecainide)	MMP-2	The inhibition of CAFs-mediated ECM deposition and mechanical remodeling also resulted in a significant reduction in gel contraction of CAFs, further eliminating the stem cell-promoting effects of CAFs	Doldi et al. (2024)

CAFs, cancer-associated fibroblasts; PCSCs, prostate cancer stem cells; AR, androgen receptor; FlnA, Filamin A; YAP1, yes-associated protein 1; TEAD1, TEA domain transcription factor 1; NFs, normal fibroblasts; PNP, protein nanoparticles; CXCL12, chemokine (C-X-C motif) ligand 12; mAb, monoclonal antibody; FAP-α, fibroblast activation protein-α; Nox4, NADPH oxidase 4; TGF-β1, transforming growth factor-β1; GSH, glutathione; ROS, reactive oxygen species; PERK, proline extensin-like receptor kinase; eIF2 α, eukaryotic initiation factor-2α; ATF4, activating transcription factor 4; MAOA, monoamine oxidase A; mTOR, mammalian target of rapamycin; HIF-1α, hypoxia-inducible factor-1α; EMT, epithelial-mesenchymal transition; CXCR4, C-X-C chemokine receptor type 4; IL-6, interleukin-6; STAT3, signal transducer and activator of transcription 3; MMPs, matrix metalloproteinases; PDGFRα, platelet-derived growth factor receptors; ECM, extracellular matrix remodeling.

### 5.2 Targeting CAF-derived factors to circumvent stemness effects

CAF-derived factors activate key pathways in PCa cells and mediate the CSC phenotype. These factors, including SDF-1, TGF-β, and HEF, trigger the development of CSC phenotypes through the PI3K/AKT pathway. However, the PI3K inhibitor (BKM120) effectively suppresses PCSCs (Wang L. et al., 2019). Moreover, the PI3K inhibitor (LY294002) effectively suppresses LNCaP and LNCaP/CAF spheroid proliferation (Eder et al., 2016).

Tumor-derived IL-6 stimulates the STAT3 pathway in CAFs (Karakasheva et al., 2018), sustaining the CSC-like characteristics of tumor cells by prompting CAFs to release MMPs (Liao et al., 2017). IL-6 signaling creates an appropriate environment for stimulating CD44 expression. Conversely, blocking the IL-6/STAT3 signaling pathway hinders CD44 expression as well as the acquisition of CSC-like characteristics and aggressive tumor behavior (Wu et al., 2019). MMPs function as mediators of IL-6/STAT3 signaling in PCa. The MMP inhibitor ilostat inhibits CAF-mediated CSC-like characteristics in PCa cells (Giannoni et al., 2011). Other inhibitors targeting IL-6/STAT3 signaling have shown antitumor effects in preclinical studies (Johnson et al., 2018). Mitoxantrone/prednisone in combination with or without cetuximab (CNTO 328) (Fizazi et al., 2012), an anti-IL-6 chimeric monoclonal antibody, was evaluated in an open-label phase II trial in patients with desmoplasia-resistant PCa who had received prior cetuxetaxel-based chemotherapy; however, the combination did not improve clinical outcomes.

CXCL12 and its receptor, CXCR4, contribute to stem cell homing and metastasis. CXCL12 induces migration of CD133+ RC-92a/hTERT cells co-expressing CXCR4, which can be inhibited by an anti-CXCR4 antibody (Miki et al., 2007). Activation of the SDF-1/CXCR4 pathway occurs in CD44^+^CD133+ PCSCs and affects cell adhesion, clonogenic proliferation, and tumorigenicity. However, the CXCR4 antagonist AMD3100 inhibits sphere formation and restores the chemosensitivity of PCSCs (Jung et al., 2018). AMD3100 enhanced the sensitivity of prostate cancer to docetaxel treatment in a preclinical model of prostate cancer bone metastases, demonstrating the therapeutic promise of targeting CXCR4. AMD3100 has shown potential in phase I, II, and III clinical studies for acute myeloid leukemia (Uy et al., 2012), and initial effectiveness in a phase I/II study for HER2-positive breast cancer (Lefort et al., 2017). No clinical studies have been conducted to study the effects of combining CXCL12 pathway inhibitors with other medicines in the treatment of prostate cancer.

MAOA plays a crucial role in regulating the stemness of PCa cells and drug resistance of stromal fibroblasts through IL-6 release and STAT3 activation in neighboring cells. Clorgyline, a MAOA inhibitor, effectively suppresses tumor development in mice, increasing caspase three levels and decreasing IL-6, pSTAT3, STAT3, and CD44 production in tumor cells (Li J. et al., 2020). In summary, CAFs maintain the CSC phenotype maintenance via the activation of derived factors and induction of key pathways. Inhibition of CAF-derived factors, such as PI3K/AKT, IL-6/STAT3, and CXCL12/CXCR4 signaling pathways, effectively suppressed PCSC phenotypes and metastatic ability (Table 2).

These findings emphasize the therapeutic potential of CAF-oriented therapy, suggesting that depleting CAFs and their derivatives may inhibit PCSC proliferation. However, further clinical research is needed to optimize this therapeutic approach.

### 5.3 Targeting the ECM

Inhibiting the formation of ECM-associated proteins or disrupting the ECM may impede its pro-tumorigenic effects and alter its stiffness, thereby disrupting the ecological niche of PCSCs and allowing drugs to exert their effects. Proteomic investigations revealed elevated expression of lysyl oxidase-like 2 (LOXL2) in CAFs. LOXL2, a copper-dependent amine oxidase, facilitates the formation of covalent crosslinks between collagen and elastin in the ECM. Therefore, administering the LOXL2 inhibitor PXS-S2a can reverse the alignment of the ECM formed by CAFs to that of regular fibroblasts, reducing the invasiveness of cocultured RWPE-2 cells (Nguyen et al., 2019). The LOX inhibitor PXS-5505 has entered Phase II clinical trials for the treatment of bone cancer myelofibrosis (Schilter et al., 2019).

Ji et al. (2016) developed a drug delivery nanosystem targeting CAFs using a cleavable amphiphilic peptide (CAP) that selectively reacts with FAP-a. In solution, CAP self-assembles into fibrous nanostructures that transform into spherical nanoparticles (NPs) upon the encapsulation of hydrophobic drugs. Upon entering the tumor stroma, these CAP-NPs were cleaved by FAP-a to efficiently release the encapsulated drug at the tumor site. This strategy breaks the stromal barrier and enhances tumor targeting and drug delivery.

Compared to normal fibroblasts, CAFs produce fibronectin-rich ECM, mediated by α5β1 integrins and PDGFRα, increasing CAF contractility and traction, and promoting PCa cell migration. Meanwhile, blocking PDGFRα activity with a neutralizing antibody, AF307, decreased integrin α5β1 expression, reducing the traction forces exerted on fibronectin and decreasing the contractile capacity of CAFs. Thus, the stromal organization of CAFs changes from aligned fibers to a random organization (Erdogan et al., 2017). Therefore, targeting or degrading the ECM presents a promising therapeutic strategy. Altering ECM organization and stiffness may disrupt the ecological niche of PCSCs, thereby facilitating drug delivery (Table 2).

According to a recent research, antiarrhythmic medicines (amiodarone, verapamil, nifedipine, flecainide) hinder the capacity of CAFs to modify the extracellular matrix via influencing cation channels. These drugs specifically reduced the ability of CAFs to move by interfering with the formation of focal adhesions. Additionally, the drugs significantly inhibited the secretion of Col1a1 and fibronectin in the surrounding environment. This not only prevented CAFs from depositing and remodeling the extracellular matrix, but also caused a significant decrease in CAF gel contraction. Subsequent research indicated that the suppression of extracellular remodeling by antiarrhythmic medications may be linked to a decrease in the production of MMP-2 by CAFs. Remarkably, the administration of antiarrhythmic drugs largely eradicated the stem cell-enhancing effects of cancer-associated fibroblasts (CAFs), with a notable impact on CD133. Nevertheless, the impact on CD44 was only found while using amiodarone (Doldi et al., 2024).

## 6 Application of organoid models in CAFs/PCSCs

The absence of appropriate *in vitro* models has hindered research on the relationship between CAFs and PCSCs. Three-dimensional (3D) organoid culture models comprise isolated multipotent stem cells or organ precursor cells that are embedded in a matrix. These models preserve the diverse characteristics and genomic properties of CSCs. Additionally, they accurately mimic the TME by maintaining close contact between CSCs and microenvironmental matrices, primarily CAFs. This enables researchers to study the communication and interactions between CSCs and their microenvironment (Lau et al., 2020).

Organoid culture is a technique used to grow primary tissues, embryonic stem cells, and induced pluripotent stem cells in a 3D environment using ECM. Although their composition is similar to that of sphere cultures, organoid media are more specific for culture targets, facilitating phenotypic observation through specific growth factors (Fatehullah et al., 2016), and can closely reproduce the *in vivo* environment (Rookmaaker et al., 2015). Hence, organoids derived from primary benign cells can be used in conjunction with regenerative and translational models to study organ development, tumor cell origins, and mechanisms underlying cancer development. In a pioneering organoid study, epithelial organoids were successfully created using LGR5+ stem cells. However, to achieve long-term organoid culture, myriad niche factors (Wnt-3A, R-Spondin, EGF, and Noggin) are required to preserve cell stemness and promote proliferation (Sato et al., 2009). Gao et al. (2014) made initial progress in established organoid models of PCa by culturing seven subtypes from biopsy specimens and circulating tumor cells. Additional research has indicated that individual luminal stem/progenitor cells obtained from CARN (castration-resistant NKX3.1-expressing cells) can produce prostate organoids in matrix-free 3D culture that exhibit functional AR signaling (Chua et al., 2014). However, most PCa organoid studies have been performed using patient-derived models that are predominantly epithelial and lack relevant mesenchymal components (Beshiri et al., 2018; Puca et al., 2018). Epithelial-mesenchymal interactions are crucial for PCa advancement and stemness (Lee et al., 2016; Mei et al., 2019). CAFs, immune cells, and vascular endothelial cells contribute to epithelial-mesenchymal transformation (Hanahan and Weinberg, 2011). CAFs cocultured with prostate epithelial cells alter epithelial cell characteristics, conferring tumor stem cell properties (Clark et al., 2013). Incorporating prostate stromal cells into organoid systems has resulted in higher viability and transmissibility (Richards et al., 2019). Liu et al. (2012) used a Rho kinase inhibitor (Y-27632) and fibroblasts as feeder layers to stimulate cell proliferation. During conditional reprogramming, tumor cells undergo culture while maintaining the cell proliferation phase and the expression of markers associated with luminal and stem cells. Based on this premise, different stromal components and growth factors, such as FGF10, FGF2, and prostaglandin E2 (PGE2), can be added to obtain a mature organoid model of PCa, forming a glandular structure with a stable karyotype similar to that of PCa *in vivo* and complete AR signaling (Drost et al., 2016). These findings confirm the importance of stromal components for maintaining the malignant characteristics of PCa cells. However, integrating stromal components into prostate organ cultures remains challenging. Therefore, the discovery of culture conditions that favor CAFs with PCSCs is a key breakthrough in organoid models. Other related issues include i) the ratio of different CAF phenotypes to PCSCs, ii) functional heterogeneity of CAFs that remodel the ECM, and iii) ECM stiffness.

**FIGURE 2:**
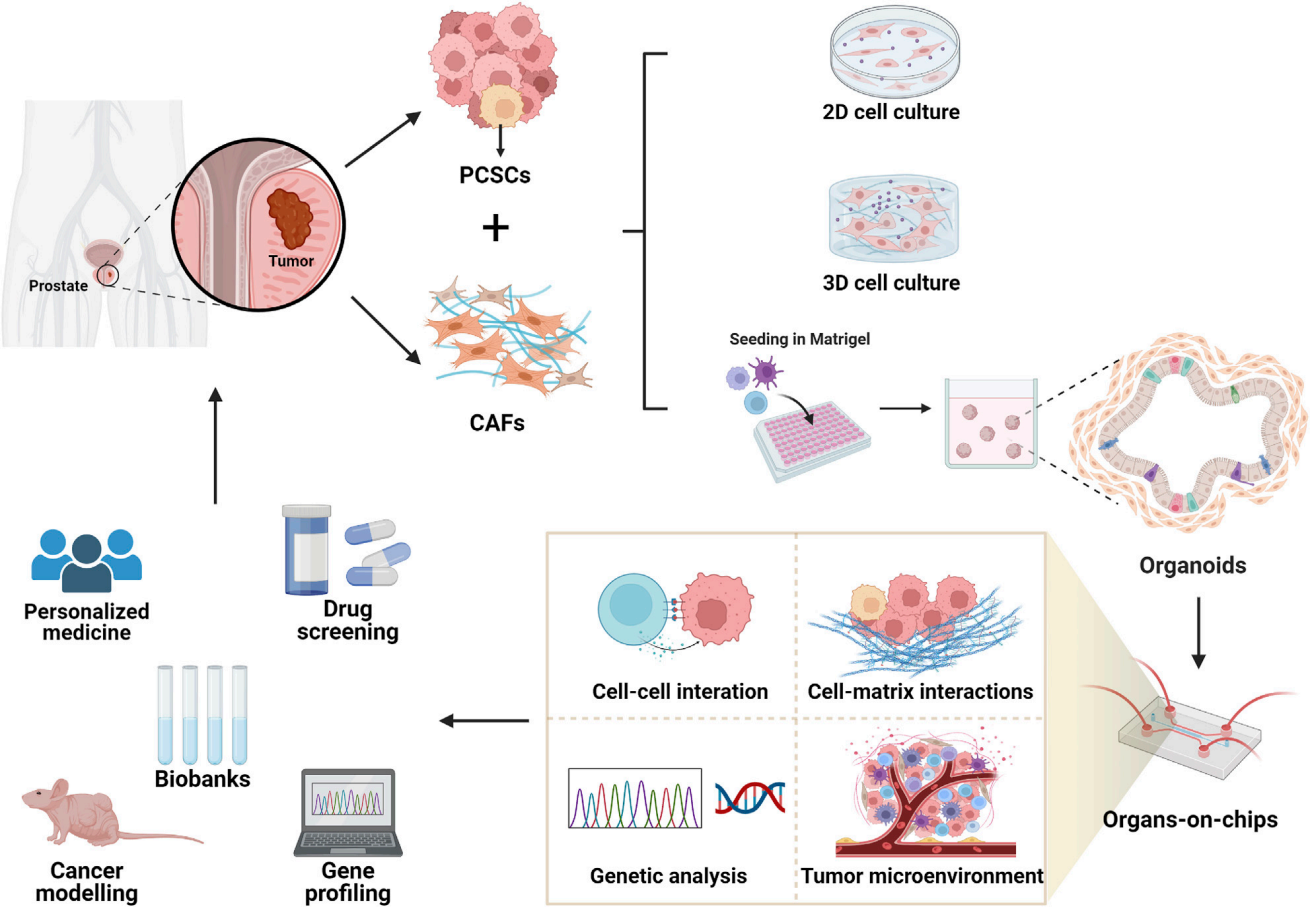
Organoids and organ-on-a-chip technology. Compared with 2D and 3D coculture systems, organoids better recapitulate the tumor microenvironment and more intuitively represent the stimulatory or maintenance effects of CAFs on PCSCs. By incorporating organ-on-a-chip technology, organoid cultures were used to pair multiple components of the microenvironment. This approach aims to facilitate the exchange of information between components in a research microfluidic system and to enable controlled and reproducible organoid cultures. The combination of 4D imaging allows dynamic monitoring of cell-to-cell and cell-to-ECM interactions, and the overall layout of the tumor microenvironment. This technology probes the crosstalk between components from a genetic analysis perspective, holding important research value for modeling, drug screening, gene profiling, and personalized targeted therapy. Created using Biorender.com.

Recently, organoid cultures have been used to pair multiple components in a microenvironment by incorporating organ-on-a-chip technologies. This method seeks to facilitate the study of information exchange between components in a microfluidic system and to achieve controlled and reproducible organoid culture. The combination of 4D imaging technology enables dynamic monitoring of the interactions between CAFs and PCSCs, which is important for studying the dynamics of coculture systems (Ao et al., 2020). Hence, tumor organoid cultures combine cell biology with micromachining and microfluidic devices. Organoids cultured in 3D bionic matrices can also co-cultivate tumor, stromal, and immune cells (Paggi et al., 2022). The cost-effectiveness of organ-on-chip technology is expected to facilitate efficient drug testing on a large scale (Sontheimer Phelps et al., 2019). This technology is expected to have significant applications in bio-precision medicine, PCa modeling, and novel drug screening (Figure 2).

## 7 Future directions

Recently, novel therapeutic strategies targeting PCSCs and the tumor microenvironment have become popular research topics, which has led to the development and assessment of various drugs and therapeutic approaches targeting PCSCs and the tumor microenvironment in preclinical studies. However, the heterogeneity and plasticity of tumors and complex interactions between CAFs and PCSCs severely impede the clinical application of targeted therapies. Most drugs targeting a single pathway in CAFs or PCSCs have failed to significantly improve antitumor efficacy in patients (Pienta et al., 2013; Johnson et al., 2018). PCSCs can activate different CAF phenotypes and maintain self-renewal and drug resistance through different paracrine pathways. Additionally, the absence of dependable biomarkers for predicting treatment responses may contribute to the unsuccessful clinical studies targeting CAFs and PCSCs. Therefore, a combination therapy targeting multiple CAF/PCSC pathways is required to enhance their antitumor activity.

## 8 Conclusion

PCSCs are key drivers of PCa progression, playing dominate roles in tumorigenesis, metastasis, drug resistance, and recurrence. During tumor formation, PCSCs create a tumor microenvironment conducive to their own survival by continuously recruiting and activating surrounding stromal cells. CAFs can regulate PCSCs by secreting cytokines, and PCSCs can secrete corresponding molecules to regulate the phenotypic transformation of CAFs, forming a feedback loop to further promote tumor progression. The interplay between CAFs and PCSCs is crucial for the progression of PCa. Investigating these interactions within the tumor microenvironment and cancer cells can help to elucidate the mechanisms underlying PCa development and establish a theoretical foundation for designing novel therapeutic approaches. Moreover, the continuation of such research may lead to the development of personalized therapies for patients with PCa, thereby providing novel targeted techniques designed to prevent tumor metastasis and recurrence.
